# Supplementary material for: Patterns of Intron Gain and Loss in Fungi
Source: PLoS Biol. 2004 Nov 30;2(12):e422. doi: 10.1371/journal.pbio.0020422 (PMC532390; doi:10.1371/journal.pbio.0020422)
Supplement: Table S1 — Also available at http://genes.mit.edu/NielsenEtAl/. (4.3 MB ZIP). [file pbio.0020422.st001.zip › NielsenEtAl/html/436.html]

AN6182.1.NCU04460.1.MG05098.1.FG05668.1


```
 CLUSTAL W (1.82) Multiple Sequence Alignments - Introns Inserted


Sequence 1: NCU04460.1	392 aa
Sequence 2: FG05668.1	382 aa
Sequence 3: MG05098.1	384 aa
Sequence 4: AN6182.1	382 aa
Alignment Length: 392 aa
Number Identitical Residues: 217 aa
Alignment Score (without introns) 10815


MG05098.1 	MPDKILDDISHRRYNPLTGTWLLVSPHRTKRPWQ~GQQEAPSKSELPQYDPS~CYLCPGN
NCU04460.1	MTDKVLDDISHRRYNPLNGSWLLVSPHRTKRPWQ2GQQEAPALNKLPEYDPQ0CYLCPGN
FG05668.1 	MPDQILDDISHRRYNPLTDSWLLVSPHRTKRPWQ2GQQEAAVATELPEYDPK0CYLCPGN
AN6182.1  	MVESVLDDISHRRYNPLRGAYVLVSPHRTKRPWQ2GAQETPSKTTLPDYDAT~CYLCPGN
          	* :.:************ .:::************ * **:.  . **:**.  *******

MG05098.1 	TRAQGDSNPKYEKTFVFVNDYSAVKETQAHYAPEEKTD~-----D1VASLLLKADAATGR
NCU04460.1	KRAQGDSNPHYKNTFAFVNDYSAVKEEQQEYHPRKDAA~GDGDDD~IASLLLQAQPATGR
FG05668.1 	PRAAGDSNPKYERTFAFVNDYSAVKQEQPEYDAKASSN~-----D~LESLLLQAQGVKGV
AN6182.1  	KRAQGDHNPKYEKTFIFVNDYSAVKEEQAPYHPEAGTE1------~TESFFLRADPVTGK
          	 ** ** **:*:.** *********: *  * ..  :           *::*:*: ..* 

MG05098.1 	CYVLTFSAVHNVTLADMKPQEIVPVIDLWTDLYASHVCPTSPLGTAAATVRQKLSIVPEC
NCU04460.1	CYVLTFSAKHDTTLADMSATEIVPVIETWTRIYASHLSASHPLRDAAARSLSEIPPNPDG
FG05668.1 	CYVLTFSPKHHVTLADMTANEIVPVIEHWTRIYANHLTPSNPR--AAEADQLNISMSKD-
AN6182.1  	CYVLTFSAAHNLTLADLSPVEIVPVINAWTDVYIAHLSPSSPL--AAAASKLTISSGSPA
          	*******. *. ****:.. ******: ** :*  *: .: *   **      :.     

MG05098.1 	-QMSQPSQQLRYMTIFENKGAAMGCSNPHPHCQIWTTSSLPEEPGKELDQMSKYRREHG-
NCU04460.1	EVEPAKKQQLRYMQIFENKGAAMGCSNPHPHCQIWTTSTLPEEPGKELAQMTKYHREHKG
FG05668.1 	-TTPSPEEQYRYMQIFENKGAAMGCSNPHPHCQVWTTSTMPEEPGKELVQMTKYREQHEG
AN6182.1  	ASLAKPNEQYRYMQIFENKGAAMGCSNPHPHGQIWTTSSLPEELAAELEQMKKYRREHNG
          	   .  .:* *** ***************** *:****::*** . ** **.**:.:* .

MG05098.1 	RHMLGDYVKLEIEKGERVVWQNDSFLIVCPWWAVWPFEVLVLAKRHVRALVDLSTDERLH
NCU04460.1	RHLLEDYVKVEMAKGERVVWQNDGFLVVCPWWAVWPFEVLVIAKRHVRALVELTSEERLQ
FG05668.1 	RHLLGDYVKLELEKKERVVWENDAFVIVCPWWAVWPFEVLVLPKRHIRSLLDFKPEERLQ
AN6182.1  	GHMLADYAALESKKQERVVFENDAFLVVCPWWAIWPFETMIISKTHKRALVDLDDNEKAQ
          	 *:* **. :*  * ****::**.*::******:****.:::.* * *:*:::  :*: :

MG05098.1 	FAEAIQEVTRRYDNLFETNFPYS1SGIHQAPIDCSEEEAESSWFHMHFYPPLLRSATVKK
NCU04460.1	FAEAVQEVTRRYDNLFETNFPYS1SGIHQAPLDCTEEEAETSWFHMHFYPPLLRSATVRK
FG05668.1 	FAEAIQEVTRRYDNLFECNFPYS~SGLHQAPLDGTPEELESAYFHMHFYPPLLRSATVKK
AN6182.1  	LAEAIAEVTRRYDNLFETHFPYS~MGIHQAPLEGSEEEIEASYLHLHFYPPLLRSATVRK
          	:***: *********** :****  *:****:: : ** *::::*:************:*

MG05098.1 	FLVG~YELLAEPQRDITPEQAAARLRACGGE~LYRNSL-
NCU04460.1	FLVG~YELMAEPQRDITPEQAAARLRDCGGE~LYRKSLQ
FG05668.1 	FLVG~YELLAEPQRDITPEQATVRLRNCGGE~LYRKSL-
AN6182.1  	FLVG2YELMAEPQRDITPEQAAARLRGCG-E1VDDAHE-
          	**** ***:************:.*** ** * :
```
